# Cerebellar cognitive affective syndrome: insights from Joubert syndrome

**DOI:** 10.1186/s40673-018-0085-y

**Published:** 2018-03-21

**Authors:** Chelsea L. Hickey, Janet C. Sherman, Paula Goldenberg, Amy Kritzer, Paul Caruso, Jeremy D. Schmahmann, Mary K. Colvin

**Affiliations:** 1 Departments of Psychiatry, Massachusetts General Hospital, Boston, MA 02114 USA; 2 Departments of Genetics, Massachusetts General Hospital, Boston, MA 02114 USA; 3 Division of Genetics and Genomics, Boston Children’s Hospital, Boston, MA 02115 USA; 4 Departments of Radiology, Massachusetts General Hospital, Boston, MA 02114 USA; 5 Departments of Neurology, Massachusetts General Hospital, Boston, MA 02114 USA

**Keywords:** Cerebellum, Joubert syndrome, Cognition, Behavior, Neuropsychology

## Abstract

**Background:**

Joubert syndrome (JS) is a rare, autosomal recessively inherited genetic disorder characterized morphologically by unique developmental malformations of the cerebellum and brainstem (the molar tooth sign), and clinically by impaired motor functions and intellectual disability. Patients with JS often face multiple cognitive challenges, but the neuropsychological profile of this condition has not been well characterized.

**Methods:**

We performed comprehensive neurological and neuropsychological evaluations in three adult brothers with JS, ages 32, 27, and 25 years.

**Results:**

They all exhibited impaired motor control, global developmental delay most evident in executive function, affect regulation, and social skill set, and similar patterns of neuropsychiatric symptoms.

**Conclusions:**

These findings provide new insights into the intellectual and neurobehavioral phenotype of JS, which we regard as a developmental form of the cerebellar cognitive affective / Schmahmann syndrome (CCAS). These observations have direct clinical relevance for the diagnosis and care of patients with JS, and they help further the understanding of the multiple manifestations of atypical cerebrocerebellar development.

## Background

**Fig. 1:**
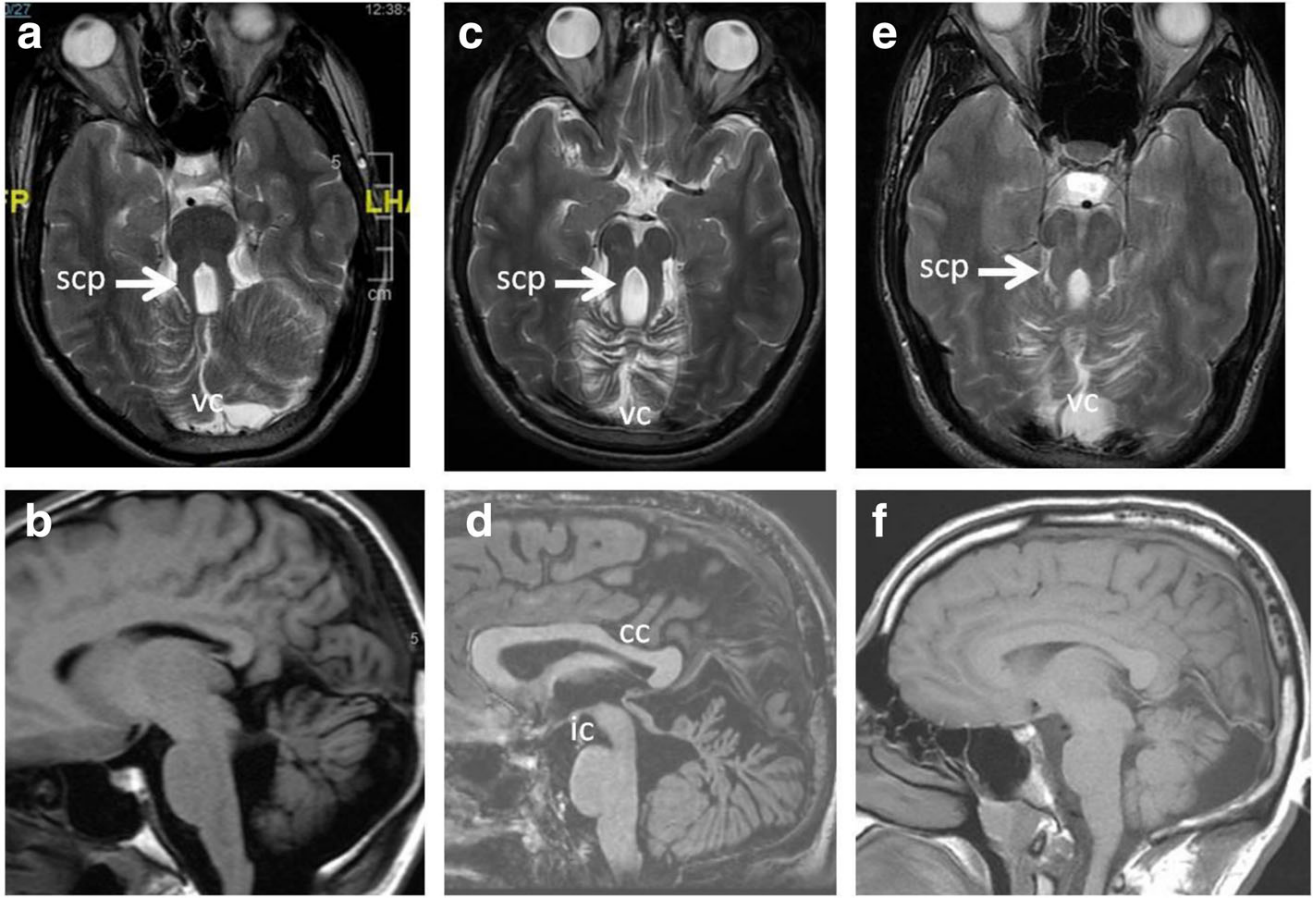
**a-f** MR images of the three patients with TMEM67 mutations/Joubert syndrome 6 phenotype. **a** an axial T2, and (**b**), a sagittal T1 weighted images in case 1, show the elongated superior cerebellar peduncles (scp, white arrow) that exhibit a classic molar tooth configuration, vemian and cerebellar hemispheric dysplasia, and a characteristic midline vemian cleft (vc). **c** an axial T2, and (**d**) a sagittal T1 weighted images in case 2, show the elongated superior cerebellar peduncles (scp, white arrow), vemian dysplasia, a characteristic midline vemian cleft (vc), deep interpeduncular cistern (ic), and an unusually contoured corpus callosum (cc). **e** an axial T2, and (**f**) a sagittal T1 weighted images in case 3, show the elongated superior cerebellar peduncles (scp, white arrow), vemian and cerebellar hemispheric dysplasia, and the characteristic midline vemian cleft (vc)

Joubert syndrome (JS) is an autosomal recessively inherited genetic disorder caused by ten currently known genes. It occurs with a 2:1 male to female ratio, and is characterized by unique morphological, systemic, and neurological manifestations [1]. Hypoplasia or malformation of the cerebellar vermis, thickened and elongated superior cerebellar peduncles, and abnormally deep interpeduncular fossa and enlarged fourth ventricle [2] together constitute the pathognomonic molar tooth sign in axial (horizontal) sections on brain imaging (Fig. 1). Brainstem and cerebral cortical atrophy, and delayed myelination may also occur [3]. Systemic abnormalities include respiratory difficulty with irregular breathing or hyperpnea in the neonatal period, renal cysts and nephronophthisis, congenital hepatic fibrosis, and ocular colobomas [2, 4–6]. The neurology of JS is usually defined by oculomotor apraxia, the cerebellar motor syndrome (ocular and extremity dysmetria, gait ataxia, dysarthria and hypotonia), and psychomotor delay [2, 4, 5].

Whereas one report described an individual with normal cognitive function [7], developmental delay is generally a key feature of JS, with impairments across several cognitive and affective domains. Full Scale Intelligence Quotient (IQ) scores range from severe intellectual disability, IQ < 30, to low average cognitive ability, IQ 85 [8, 9]. The few neuropsychological studies performed in JS to date document impairments in verbal memory, executive function, verbal fluency, visuomotor integration, and fine motor control [10, 11], with neuropsychiatric features including hyperactivity, aggression, and mood regulation [10]. This constellation of symptoms has not been more fully characterized, however, nor linked to the neuroanatomical abnormalities.

It is now apparent that the cerebellum is integrally involved in the regulation of intellect and emotion [12–14]. Anatomical substrates defined in animal tract tracing studies link cerebral association areas with the cerebellum in reciprocal feedforward and feedback loops [15, 16]. Functional topography in the human cerebellum has been identified for movement, cognition and social-emotional processing using resting state [17] and task-based [18, 19] functional magnetic resonance brain imaging studies. Lesion-deficit correlation studies in patients reveal that lesions in the cerebellar anterior lobe cause the cerebellar motor syndrome [20–22], whereas lesions of the cerebellar posterior lobe are responsible for the cerebellar cognitive affective syndrome (CCAS; [23–25]; Schmahmann syndrome; [26]). The CCAS deficits are concentrated in the domains of executive control, visuospatial processing, and linguistic functions. Executive impairments are seen in planning, set-shifting, abstract reasoning, working memory, multitasking and verbal fluency; spatial reasoning problems include higher-order aspects of visuospatial processing; language errors manifest as agrammatism, anomia, and aprosodia; and patients also have difficulties with mental arithmetic and unstructured recall of newly learned information [23]. Affective impairments, the neuropsychiatry of the cerebellum, manifest as impaired modulation of emotions and behavior, and segregate into five domains - attentional control, emotional control, autism spectrum, psychosis spectrum, and social skill set, each of which includes symptoms of excessive (positive) or reduced (negative) behaviors [27]. The CCAS has been widely replicated in adults and children with acquired and neurodegenerative cerebellar diseases [28], as well as in those with congenital or neonatal injury, leading to the concept of developmental CCAS [27, 29–33].

Cerebellar dysmorphology is at the core of the neurology of JS, and we therefore hypothesized that the neuropsychology and neuropsychiatry of JS should align with prior reports of these deficits in patients with cerebellar pathology. Here we test this hypothesis in three brothers with JS by performing comprehensive neurological and neuropsychological evaluations to characterize their cognitive, emotional and behavioral presentations, and to compare and contrast them with each other and with the published accounts of developmental CCAS.

## Methods

We studied three biological brothers, all left-handed Caucasians of middle-socioeconomic status, who were diagnosed with JS by clinical, radiographic and molecular genetic testing: Case 1 (32 years of age), Case 2 (27 years), and Case 3 (25 years). All underwent a comprehensive neuropsychological evaluation within weeks of each other, supervised by a licensed and board-certified neuropsychologist (MKC). Medical records were reviewed, including salient historical facts, medications, general and neurological examination findings, and results of brain imaging investigations and genetic testing. Neuropsychological evaluations consisted of assessment of cognition (Wechsler Adult Intelligence Scale-Fourth Edition (WAIS-IV; [34]), Repeatable Battery for Neuropsychological Status (RBANS; [35]), academic abilities (Wechsler Individual Achievement Test (WIAT-III; [36]), motor control (Grooved Pegboard test (GP; [37]), and maternal report of adaptive functioning (Scale of Independent Behavior-Revised (SIB-R; [38]). The incidence of neuropsychiatric symptoms was assessed through qualitative analysis of records, and through standard clinical psychiatric assessment interviews carried out with each patient and their mother as part of the neuropsychological evaluation. Questions were targeted towards determining the presence and severity of neuropsychiatric symptoms, including mood, anxiety, thought disorder, social communication, inattention, and externalizing behaviors consistent with criteria of the DSM-V. Structured questionnaires were not administered to the patients because of their reading level and cognitive variability. This study was approved by the Institutional Review Board of the Massachusetts General Hospital. Written, informed consent was obtained from all participants and their legal guardian to perform this study and to present their deidentified medical information in this report.

## Results

### Genetic and neurological features

All three brothers were compound heterozygotes for pathogenic mutations of *TMEM67* (c.245 C > G p.Pro82Arg; c.755 T > C p.Met252Thr), confirming their clinical diagnosis. At the time of evaluation in adulthood they all displayed the ophthalmological finding of coloboma; alternating exophoria and nystagmus, dysarthria with impaired articulatory clarity and reduced prosody. Case 3 had lower extremity spasticity and hyper-reflexia but flexor plantar responses, stereotypies of the hands, head and neck, and mildly slow and clumsy movements of the arms and legs. Cases 1 and 2 had wide-based gait with pronation; case 3 had a narrow-based gait with occasional scissoring, and extra steps to turn accompanied by rolling movements of his neck and upper torso. All three had a laboratory-confirmed diagnosis of sleep apnea. The patterns of malformations on brain MRI for all three patients were diagnostic of JS (see Fig. 1), with a deep interpeduncular cistern, dysplastic cerebellar vermis and elongated superior cerebellar peduncles. In addition, Case 2 had thinning of the corpus callosum particularly at the posterior body and isthmus.

### Developmental and medical histories

The three brothers demonstrated varying levels of developmental delays. All received special education services with placement in substantially separate learning environments.

Case 1, the oldest brother, was born full term and managed in the neonatal intensive care unit (NICU) for irregular breathing, although no respiratory interventions were required. He had subarachnoid hemorrhage (location not further specified in the medical record) with macrocephaly, but he did not require a ventricular shunt. Motor and language milestones were significantly delayed. He sat at 18 months, walked with a walker at 3 years, and walked independently at 5 years. He spoke his first words at 18–20 months and started combining words at 2 years. He received special education services throughout school, a high school diploma at age 20 and completed 1 year of undergraduate education. At the time of our evaluation, he was employed performing janitorial services. He had incidental inguinal hernia, diverting ileostomy, and chronic sinusitis. Concussion at age 18 in a motor vehicle accident was associated with no new neuroimaging findings although he experienced transient worsening of his ataxia and cognitive symptoms.

Case 2, the middle brother, was born full term without perinatal complications. He sat at 24 months, walked with a walker at 3.5 years, and walked independently at 5 years. He initially used sign language for communication and began combining words at 4 years. He received interventions including physical therapy, occupational therapy, and speech therapy throughout his childhood. There were recurrent upper respiratory and ear infections in childhood. He received special education services throughout school and received a certificate of completion at age 22. At the time of our evaluation, he was enrolled in a day program focusing on developing social and functional skills.

Case 3, the youngest brother, was born full-term and managed in the NICU for tachypnea, although no respiratory interventions were required. He required nutritional supplements in infancy to promote weight gain, and was found to have congenital hepatic fibrosis. He sat at 2 years, walked with a walker at 5 years, and walked independently at 7 years. He initially used sign language to communicate and began speaking at 6 years. He received special education services throughout school and received a certificate of completion at age 22. At the time of our evaluation, Case 3 was not engaged in vocational activities or attending a day program.

All three brothers lived at home with their parents.

### Neuropsychological evaluations

The three patients all performed below age-based expectations across cognitive, academic, and adaptive skills domains. Detailed results are presented in Table 1. Given their developmental history and adaptive functioning, Case 1 (WAIS-IV, Full Scale IQ = 67) was diagnosed with Borderline Intellectual Functioning. Although a WAIS-IV FSIQ of 67 would typically reflect Mildly Impaired Intellectual Functioning, Case 1 came to the evaluation with a prior diagnosis of Borderline Intellectual Functioning due to his higher level of adaptive functioning, and this diagnosis was retained. Cases 2 (WAIS-IV, Full Scale IQ = 53) and 3 (WAIS-IV, Full Scale IQ = 59) had diagnoses of Intellectual Disability. Cases 1 and 2 demonstrated generally commensurate verbal and nonverbal intellectual abilities. Case 3 demonstrated stronger nonverbal intellectual skills (WAIS-IV, Perceptual Reasoning Index = 75) relative to his verbal intellectual skills (WAIS-IV, Verbal Comprehension Index = 61). This contrasts with most investigations to date which have documented a stronger verbal than visuoperceptual abilities in individuals with cerebellar disorders [23, 39].

**Table 1:** Scores on Neuropsychological Testing

	Case 1		Case 2		Case 3	
	SS	%ile	SS	%ile	SS	%ile
WAIS-IV Verbal Comprehension Index	76	5	63	1	61	< 1
Similarities	6	9	3	1	1	< 1
Vocabulary	5	5	4	2	4	2
Information	6	9	4	2	5	5
WAIS-IV Perceptual Reasoning Index	73	4	60	< 1	75	5
Block Design	6	9	3	1	6	9
Matrix Reasoning	5	5	3	1	5	5
Visual Puzzles	5	5	4	2	6	9
WAIS-IV Working Memory Index	71	3	58	< 1	63	1
Digit Span	6	9	3	1	3	1
Arithmetic	4	2	2	< 1	4	2
WAIS-IV Processing Speed Index	65	1	56	< 1	59	< 1
Symbol Search	4	2	2	< 1	3	1
Coding	3	1	2	< 1	2	< 1
WAIS-IV Full Scale IQ	67	1	53	< 1	59	< 1
WIAT-III						
Word Reading	78	7	61	< 1	51	< 1
Pseudoword Decoding	84	14	59	< 1	52	< 1
Numerical Ops	84	14	62	1	64	1
Spelling	86	18	65	1	56	< 1
RBANS						
Immediate Memory						
List Learning	7	16	2	< 1	7	16
Story	7	16	2	< 1	2	< 1
Delayed Memory						
List Recall	Raw = 4		Raw = 0		Raw = 8	
List Recognition	Raw = 20		Raw = 20		Raw = 20	
Story Recall	9	37	5	5	4	2
Figure Recall	3	1	2	< 1	2	< 1
Visuospatial						
Figure Copy	1	< 1	1	< 1	1	< 1
Line Orientation		10–16		3–9		26–50
Language						
Picture Naming		17–25		51–75		3–9
Semantic Fluency	7	16	1	< 1	2	< 1
Attention						
Digit Span	8	25	4	2	2	< 1
Coding	1	< 1	1	< 1	1	< 1

*WAIS-IV* Wechsler Adult Intelligence Scale-Fourth Edition, *WIAT-III* Wechsler Individual Achievement Test-Third Edition, *RBANS* Repeatable Battery for the Assessment of Neuropsychological Status

Within the context of global developmental delay, all three patients demonstrated impairments in the domains of executive functions, visuospatial processing, and language (Table 2). In the executive domain, the three brothers were similarly impaired on processing speed (WAIS-IV Processing Speed Index) and Cases 2 and 3 were impaired on verbal fluency (RBANS Semantic Fluency). Notably, Case 2 demonstrated the greatest vulnerability across the range of executive functions. In the visuospatial domain, all three had difficulty with higher-level aspects of part-to-whole analysis and organization of spatial information, in contrast to their ability to accurately perceive basic line orientation. The visuospatial deficits were out of proportion and not attributable to their difficulties with graphomotor control. Qualitatively, all three patients had difficulties with articulation and their speech intonation patterns were atypical. On tests of language, Case 3 demonstrated a degree of anomia, while confrontation naming was an area of relative strength for Cases 1 and 2 consistent with their findings of low average to average performance (RBANS Picture Naming).

**Table 2:** Impaired Neuropsychological Performance Across Cognitive Domains Impacted in CCAS

	Case 1	Case 2	Case 3
Executive Functioning			
Set-shifting	+		+
Abstract reasoning		+	
Working memory		+	+
Verbal fluency		+	+
Processing speed	+	+	+
Visuospatial Functioning			
Line orientation			
Visuospatial organization	+	+	+
Language			
Prosody	+	+	+
Naming			+
Agrammatism		+	+

+ Represents impaired performance defined as scaled score ≤ 4 (2nd %ile)

Performance across measures of verbal memory were variable. On the RBANS List Learning task, Cases 1 and 3 demonstrated memory performances consistent with their level of intellectual functioning, including the encoding and retrieval of verbal information. Case 2 showed a significant weakness in both encoding and retrieval of verbal information. For all three patients, delayed recognition of the words was significantly stronger, indicating retention of new information over time.

Adaptive functioning was evaluated through informant (maternal) report on the SIB-R (Table 3). Case 1’s overall level of independent functioning was at a 12.9-year-old level. Level of adaptive functioning was much lower for Cases 2 and 3, rated between a 5.1 and 6.2-year-old level. Motor skills were rated at equivalent levels across all three cases, between a 4.1 and 6.3 years of age. Personal and community living skills were rated as least developed for Case 2 compared to his brothers, perhaps reflecting the impact of his greater cognitive vulnerabilities in domains of memory and executive functioning on functional skills.

**Table 3:** Scores on the Scale of Independent Behavior-Revised (SIB-R)

	Case 1			Case 2			Case 3		
SIB-R Subscale	AE	SS	%ile	AE	SS	%ile	AE	SS	%ile
Broad Independence	12.9	61	1	5.1	5	< 1	6.2	11	< 1
Motor Skills	6.3	44	< 1	4.1	16	< 1	4.5	23	< 1
Social/Communication	15.2	79	8	8.4	49	< 1	5.11	34	< 1
Personal Living	14.3	66	1	4.6	16	< 1	7	32	< 1
Community Living	15.5	69	2	6.4	6	< 1	7	14	< 1

*SIB-R* Scale of Independent Behavior-Revised, *AE* age equivalent

### Socio-emotional functioning

All three patients have a history of behavioral and emotional dysregulation. Case 1 was not diagnosed with JS until age 16 when he was evaluated for psychiatric symptoms including mood dysregulation, and auditory and visual hallucinations that resulted in multiple psychiatric hospitalizations. The JS diagnosis prompted investigations of his younger brothers leading to their diagnoses of JS at age 11 for Case 2 and age 9 for Case 3. Case 2 had depression and general anxiety disorder not necessitating psychiatric hospitalization. Case 3 was hospitalized twice at ages 23 and 24 for auditory hallucinations, delusions of grandeur, unprovoked aggression, and suicidal depression.

At the time of our evaluation, all three men endorsed emotional and behavioral symptoms that conformed to the domains of the neuropsychiatry of the cerebellum, namely attentional control, emotional control, psychosis spectrum, and social skill set (Table 4). Attentional control and social skill set was most affected, except for Case 1 who had problems predominantly with emotional control. The positive / exaggerated / hypermetric symptoms were more frequent than the negative / diminished / hypometric symptoms in Cases 1 and 2, and more evenly distributed in Case 3 who demonstrated the most neuropsychiatric symptoms.

**Table 4:** Psychiatric Symptoms Across Neurobehavioral Profile of CCAS

	Positive Symptoms		
	Case 1	Case 2	Case 3
Attentional Control			
Distractibility	+	+	+
Hyperactivity			
Emotional Control			
Impulsivity			
Mood Lability	+		
Anxiety, panic symptoms	+		
Incongruous affect	+		
Autism Spectrum			
Stereotypical behaviors			
Self-stimulation behaviors			
Psychosis Spectrum			
Illogical thought processes			+
Paranoia			+
Hallucinations			+
Social Skill Set			
Anger, aggression	+	+	+
Irritability		+	+
Oppositional behaviors			
	Negative Symptoms		
	Case 1	Case 2	Case 3
Attentional Control			
Difficulty shifting focus of attention		+	+
Emotional Control			
Sadness, depression	+		
Anhedonia			
Apathy			
Autism Spectrum			
Avoidant behaviors			
Sensory overload			
Psychosis Spectrum			
Reduced empathy			+
Muted affect			
Blunting			
Social Skill Set			
Passivity			
Difficulty interpreting social cues and pragmatics	+	+	+

+ Represents clinical presence of psychiatric symptom based on informant report and qualitative analysis by the examiner of clinical information

## Discussion

We report the case histories and neurological, cognitive and neuropsychiatric profiles of three brothers with JS and the hallmark developmental malformations of the cerebellum, each with causative homozygous pathogenic mutations in the TMEM67 gene. All three brothers had the ophthalmological finding of coloboma, and the cerebellar motor syndrome to varying degrees – nystagmus, cerebellar dysarthria with impaired articulatory clarity and reduced prosody, and unstable gait with pronation of the feet.

Neuropsychological evaluations indicated that all had global developmental delays, consistent with diagnostic criteria for intellectual disability or borderline intellectual functioning. Within this context of impairment, there were relative weaknesses in aspects of executive functions, visuospatial functions, and language functions. All three patients had slower processing speed and poor spatial organization skills, in the absence of difficulties with perceptual judgments or naming, as well as reduced verbal fluency in two of the patients. They also all had major psychiatric histories with affective instability (mood and anxiety symptoms) and in two cases, symptoms of psychosis (auditory or visual hallucinations, delusions), requiring inpatient hospitalization.

Our study is one of the first to describe the neuropsychological profile and psychiatric presentation in adults with JS. Most previous reports have focused on children [8, 10, 40]. This study further informs our understanding of the clinical manifestations of JS, with its hallmark finding of hindbrain malformation and its elementary neurological presentation with the cerebellar motor syndrome. The three siblings were all developmentally delayed, but they also demonstrated disproportionate cognitive weaknesses in selected aspects of executive function, language processing, and the visuospatial domain, as well as a pronounced neuropsychiatric constellation in each of them. This cognitive and neuropsychiatric profile is consistent with the previously described syndrome of CCAS, which may occur as an acquired or developmental disorder. Together with the cerebellar motor and vestibular syndromes, the CCAS represents the third cornerstone of ataxiology [26], the manifestation of cerebellar pathology involving the cognitive-limbic cerebellum represented in the cerebellar posterior lobe [13, 41].

The developmental histories of all three brothers were marked by prominent psychopathology: two experienced psychotic symptoms, and all were troubled by anxiety and depression. Their neuropsychiatric profile did not involve autistic-like symptoms, consistent with the conclusion that JS is distinct from autism [39, 42]. In a prospective study of 54 children, adolescents, and young adults with JS using neuropsychological and behavioral measures, 40% displayed inattention, hyperactivity, social withdrawal, and atypical behaviors whereas 7.4% of patients met clinical criteria for a conventional psychiatric diagnosis [39]. The variability in prevalence and severity of psychiatric disorders in JS may reflect the cerebellum’s unique role in the regulation of affect. Schmahmann and colleagues [27] proposed that the neuropsychiatry of the cerebellum is characterized by positive / overshoot / hypermetric symptoms and negative / undershoot / hypometric symptoms in five domains of behavior – emotional control, attentional control, psychosis spectrum disorders, autism spectrum disorders, and social skill set. The siblings in this report had a higher overall frequency of positive / overshoot symptoms than of negative / undershoot symptoms. Brain imaging revealed similar patterns of cerebellar vermis hypoplasia in all three, and it is possible therefore that vermis hypoplasia is associated with a higher frequency of positive CCAS psychiatric symptoms (e.g., aggression, distractibility, panic symptoms) than negative CCAS psychiatric symptoms (e.g., depression, apathy). This possibility is consistent with the observation by Poretti and colleagues [43] who demonstrated that a high degree of vermis hypoplasia correlates with worse neurodevelopmental outcome.

## Conclusions

We describe the motor, cognitive and neuropsychiatric phenotype in three brothers with JS. In addition to the cerebellar motor syndrome, all three brothers had varying degrees of developmental delay, with disproportionate involvement of the cognitive and neuropsychiatric domains that are the hallmark of the cerebellar cognitive affective syndrome. The nonmotor presentations were prominent, and in fact, it was the neuropsychiatric manifestations that led to diagnosis of JS in the older brother with subsequent investigations leading to the diagnoses of JS in the younger two brothers. This underscores the need for patients with JS to receive comprehensive neuropsychological and neuropsychiatric evaluations in addition to routine medical and neurological tests. Detecting and characterizing impairments and strengths in cognition and emotion in these patients facilitates rehabilitation measures that can optimize appropriate care and interventions across the lifespan. Future studies are needed to assess rehabilitation approaches to improving functional skills and quality of life in individuals with JS.

## Abbreviations

CCAS: Cerebellar cognitive affective syndrome
DSM-V: Diagnostic and statistical manual of mental disorders
GP: Grooved pegboard test
IQ: Intellectual quotient
JS: Joubert Syndrome
NICU: Neonatal intensive care unit
RBANS: Repeatable battery for neuropsychological status
SIB-R: Scale of independent behavior-revised
WAIS-IV: Wechsler Adult intelligence scale-fourth edition
WIAT-III: Wechsler individual achievement test
